# Study on TEMPO-Mediated Oxidation of *N*-Succinyl Chitosan and the Water Retention Property

**DOI:** 10.3390/molecules25204698

**Published:** 2020-10-14

**Authors:** Aoqi Li, Qinglan Xue, Yingqing Ye, Peixin Gong, Mingyu Deng, Bo Jiang

**Affiliations:** 1 College of Chemistry, Sichuan University, Chengdu 610065, China; aoqili67@163.com (A.L.); silviaxue323@163.com (Q.X.); 2 Jingkun Oilfield Chemistry Technology Development Company, Suzhou 215300, China; yeyingqing1982@126.com (Y.Y.); gpx1025@163.com (P.G.); ksdmy@vip.sina.com (M.D.)

**Keywords:** chitosan, *N*-succinyl chitosan, TEMPO, selective oxidation, water retention

## Abstract

C-6 oxidized chitosan is of great interest in obtaining a new moisture retention polymer like hyaluronic acid. The direct C-6 specific oxidation of chitosan mediated by the TEMPO/NaClO/NaBr system has proven to be difficult because of the high crystalline and high C-2 amino group content. In this work, the pre-modification of chitosan by *N*-succinylation was investigated and followed by the TEMPO-mediated C-6 specific oxidation under homogeneous conditions. The desired 6-oxidized *N*-succinyl chitosan product was obtained within 15 min with a yield of about 92%. The structure of these chitosan derivatives was confirmed by FTIR and NMR spectroscopy. Moreover, it was observed that the selective oxidation led to a great improvement in water solubility and moisture retention ability. These results present a wide range of possibilities for expanding the utilization of chitosan resources.

## 1. Introduction

In the past two decades, hyaluronic acid (HA), composed of d-glucuronic acid and *N*-acetyl-d-glucosamine, has played a critical role in cosmetics and clinical medicine because of its unique physical and chemical properties [1]. Despite the significant progress that has been achieved on the microbial production of HA, the continuous rise in the cost of raw materials weakens the commercial competitiveness of microbial HA production [2,3,4,5].

Chitosan (CS), a linear polysaccharide composed of randomly distributed β-(1→4)-d-glucosamine and *N*-acetyl-d-glucosamine, is obtained from the deacetylation of chitin, which is the second most abundant natural polysaccharide next to cellulose [6]. Because of the abundant reserves, excellent biocompatibility, and d-glucosamine as one constituent of the main chain similar to HA, CS appears to be a suitable choice for preparing a new kind of moisture retention polymer similar to hyaluronic acid. However, CS is insoluble in common solvents except acidic solution, which has severely limited its applications.

There are numerous reactive amino and hydroxyl functional groups in CS which make it easier to be modified to improve its water-solubility and extend its utilization, such as carboxylation, acylation, alkylation, and so on, where C-6 oxidized chitosan has attracted much attention [7,8,9]. Since Semmelhack et al. [10] reported that selective oxidation of primary alcohols by 2,2,6,6-tetramethylpiperidinoxyl (TEMPO) systems was feasible in the presence of secondary alcohols, TEMPO-mediated oxidation of primary hydroxyls in polysaccharide has been intensively investigated [11,12]. In the oxidation process, TEMPO was employed with a co-oxidative system, of which NaOCl/NaBr has proven to be the most commonly used system so far owing to obvious advantages such as high selectivity, high conversion rate, and low cost of co-oxidant [8,11,13]. Even so, it has been reported that the direct oxidation of CS was inefficient because of the high crystalline and high C-2 amino groups content, so pre-modifications of the substrate is often required [7,9,13]. Bordenave et al. [13] attempted to modify CS by *N*-phthaloylation, but phthaloyl groups had been removed during the oxidation stage and the oxidation was not successful. They also used *N*,*N*,*N*-trimethylation as the amino protection, which has been proven to allow the C-6 specific oxidation of chitosan, but the yield was only about 30%. Besides, Kato et al. [7] reported that when *N*-acetylated chitosans had degrees of *N*-acetylation at more than 81% as starting materials, water-soluble products could be obtained in good yields. Nevertheless, it seems that no characterization related to the moisturizing properties of the products has ever been performed. As we know, it remains challenging to carry out TEMPO-mediated oxidation of CS efficiently.

*N*-succinyl chitosan (NSCS) is a water-soluble chitosan derivative with excellent biocompatibility and biodegradability, and has been extensively studied for drug delivery, wound dressings, and biomedical materials [14,15,16,17,18]. In this paper, the C-6 selective oxidation mediated by the TEMPO/NaOCl/NaBr system was conducted under homogeneous conditions on *N*-succinyl chitosan to get a novel chitosan derivative. These derivatives were structurally characterized by FTIR, NMR spectroscopy and XRD. Furthermore, the water absorption and retention abilities were also investigated by gravimetric methods and a differential scanning calorimeter (DSC) to demonstrate the potential for expanding the utilization of CS resources.

## 2. Results and Discussion

### 2.1. Reaction Processes

**Scheme 1:**
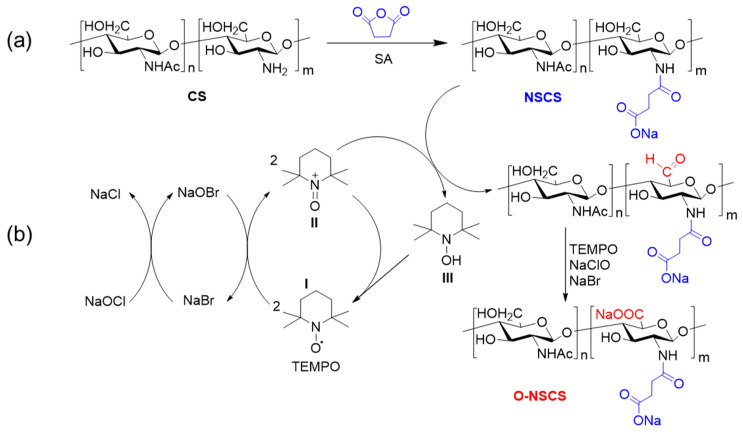
(**a**) Synthesis of *N*-succinyl chitosan (NSCS); (**b**) The oxidation mediated by the TEMPO/NaClO/NaBr system, adapted from Bragd et al. [8,20].

According to Bordenave et al. [13], the amino groups of chitosan showed an adverse effect on this oxidation reaction, which may be related to β-elimination in the alkaline reaction medium [19]. Therefore, the modified chitosan by *N*-succinylation was subjected to TEMPO-mediated selective oxidation. The integral synthesis route is shown in Scheme 1.

**Figure 1:**
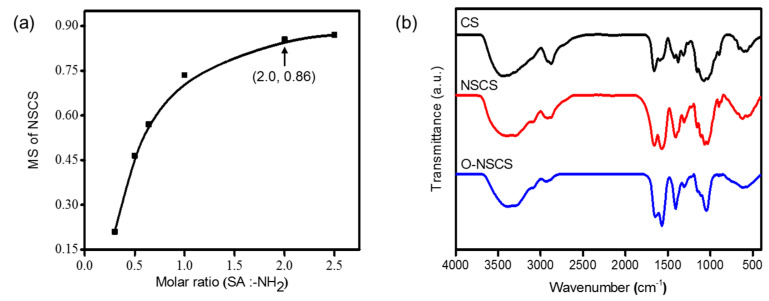
(**a**) Effect of the molar ratio of reactants on the molar substitution (MS) of NSCS. (**b**) FTIR spectra of chitosan (CS), NSCS, and 6-oxidized *N*-succinyl chitosan (O-NSCS).

NSCS was obtained by a ring-opening reaction, which was carried out under heterogeneous conditions. The reaction gave water-soluble NSCS in yields of about 95%. The desired molar substitution degree (MS) could be achieved by using a proper amount of SA following Figure 1a. The evaluation of MS will be further discussed later with ^1^H NMR spectroscopy. To minimize the negative effect of the amino group on the oxidation reaction, NSCS with MS of 0.86, produced by adding Succinic anhydride (SA) and amino group in a molar ratio of 2:1 (labeled in Figure 1a), was considered as a substrate. The Mv of NSCS mentioned above was 1350 kDa.

The regioselective oxidation mediated by the TEMPO system is a complex reaction, where TEMPO acts as a catalyst, NaBr is added as a co-catalyst, and NaClO plays the role of oxidant. The accepted catalytic cycle proceeds generally as described in Scheme 1b. The actual oxidizing species is the TEMPO^+^ ions (II) derived from oxidized TEMPO radicals (I) by NaBrO. TEMPO^+^ ions form the covalent bond with primary hydroxy groups much more preferentially than secondary hydroxy groups due to the steric hindrance. Once reacting with C-6 primary hydroxyl or aldehyde groups, II is converted into the *N*-hydroxy-TEMPO (III). TEMPO radical is continuously regenerated in situ through the reaction of one molecule of II with one molecule of III.

In this design, the reaction is fast and selective; the theoretical NaOH consumption of 100% was achieved within 15 min, and the reaction reached a yield of 92% by weight. Yoo et al. [21] claimed that 100% oxidized chitosan was produced from both specific and non-specific oxidation processes, but there was no evidence that C-6 had been completely oxidized. Bordenave et al. [13] used *N*,*N*,*N*-trimethyl chitosan as a specific oxidation substrate, but the yield was only about 30%. As we know, *N*-acetylated chitosan with degrees of *N*-acetylation of 93% was a good start material to produce a fully oxidized derivative in good yield, but it took about an hour [7]. The fast reaction rate of our design was presumably due to the low crystallinity and the corresponding high accessibility to the homogeneous TEMPO-mediated oxidation. The completely oxidized derivative was soluble in neutral water, and the Mv was 35 kDa. The depolymerizations of the chitosan are very severe, like TEMPO-mediated selective oxidation on other polysaccharides [11]. A “blank” reaction was conducted in the absence of TEMPO. The viscosity of NSCS aqueous solution decreased quickly after the introduction of NaClO, and there was almost no NaOH consumption observed, unlike the results for pure chitosan reported by Yoo et al. [21]. Thus, *N*-succinylation of chitosan may prevent the amine group from the non-selective oxidation by NaClO, but NaClO-induced oxidative degradation of the polysaccharide main chain seems inevitable.

### 2.2. Structure and Chemical Characterization of Chitosan Derivatives

#### 2.2.1. FT-IR Spectroscopy

Figure 1b represents the FTIR spectra of CS, NSCS, and O-NSCS. As shown in CS, peaks at 3500–3200 cm^−1^ were caused by the overlapped stretching vibration of O-H and N-H. Peaks at 2917 cm^−1^ and 2875 cm^−1^ resulted from the stretching vibration of C-H, and those at 1658 cm^−1^ and 1588 cm^−1^ were ascribed to the stretching vibration of C=O (Amide I) and the bending vibration of N-H (Amide II) in acetyl groups (-NH(CO)-CH_3_). Peaks at 1067 cm^−1^ and 1028 cm^−1^ represented the saccharide backbone, respectively [22].

After reacting with succinic anhydride, several peaks appeared in the spectrum compared with that of fresh CS. The new stretch at 1412 cm^−1^ was assigned to the symmetric stretching of the -COO^−^, while those peaks at 1661 cm^−1^ and 1569 cm^−1^ could be attributed to Amide I and Amide II in succinyl groups (-NH(CO)-CH_2_-CH_2_-COO^−^), respectively. Besides, there was no signal associated with the ester group at around 1730 cm^−1^, which demonstrated the successful synthesis of NSCS.

According to the FTIR spectra of NSCS, after the oxidation reaction, IR peaks at 2917 cm^−1^ and 2875 cm^−1^ due to the stretching vibration of C-H were weakened significantly, while peaks at 1569 cm^−1^ and 1412 cm^−1^ increased, which was triggered by the asymmetric and symmetric stretching vibrations of the -COO^−^. These IR results indicate that an oxidation reaction has occurred.

#### 2.2.2. NMR Spectroscopy

**Figure 2:**
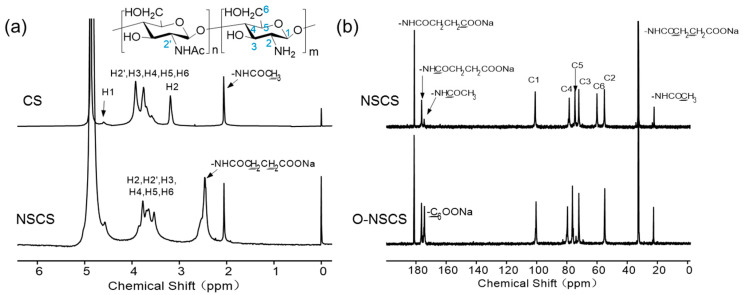
(**a**) The structure of CS and ^1^H NMR spectra of CS and NSCS with MS = 0.86 at 298K. (**b**) ^13^C NMR spectra of NSCS and O-NSCS with degree of oxidation (DO) = 100%.

^1^H NMR analysis was conducted to verify the conversion of CS in the reaction process and calculate the MS of NSCS. The ^1^H NMR spectrum of CS (in 1% DCl) was determined by Figure 2a. The resonance signal at 4.60 ppm was attributed to H_1_. The resonances for H_2_, H_3_, H_4_, H_5,_ and H_6_ on the glucose ring were found in the chemical shift range of 3.40–3.95 ppm, and the signal for H_2_ appeared at 3.19 ppm (labeled in Figure 2a). The signal at 2.06 ppm was assigned to the protons of a methyl group in acetylene (-NH(CO)-CH_3_).

Figure 2a also displays the ^1^H NMR spectrum of NSCS (in D_2_O). Compared with that of CS, the signal for H_2_ was shifted to a higher chemical shift due to less chemical shielding. There were distinctive signals at 2.47 ppm and 2.45 ppm, which were attributed to the protons of methylene groups in succinyl [14,22,23]. The ^1^H NMR result confirms the FTIR result. Besides, according to the ratio of the integral peak, the degree of molar substitution thus can be evaluated from Equation (1):

(1) $$MS=\frac{6}{4}\times \frac{I\left({\mathrm{CH}}_{2}\right)}{I\left(H_{2}\sim H_{6}\right)}$$

where *I*(CH_2_) is the integral intensity of methylene in the succinyl group; *I*(H_2_~H_6_) is the sum of integral intensities of H_2_, H_2′_, H_3_, H_4_, H_5,_ and H_6_, respectively.

^13^C NMR spectra of NSCS and its oxidized products are shown in Figure 2b. The resonance signal due to C_6_ of NSCS was detected at 60.06 ppm in the NMR spectrum, while there was no signal due to the C_6_ primary hydroxyls by the TEMPO-mediated oxidation, and the C_6_ carboxyls appeared in turn at 174.39 ppm. Moreover, the remaining resonance signals did not change significantly, and there were no chemical shifts in the range of 190–210 ppm, indicating the absence of keto groups formed by the oxidation of secondary alcohols [12]. These ^13^C NMR results suggest that selective and complete oxidation of the C_6_ primary hydroxyls of chitosan was, accordingly, achieved by using NSCS as a starting material.

#### 2.2.3. XRD

**Figure 3:**
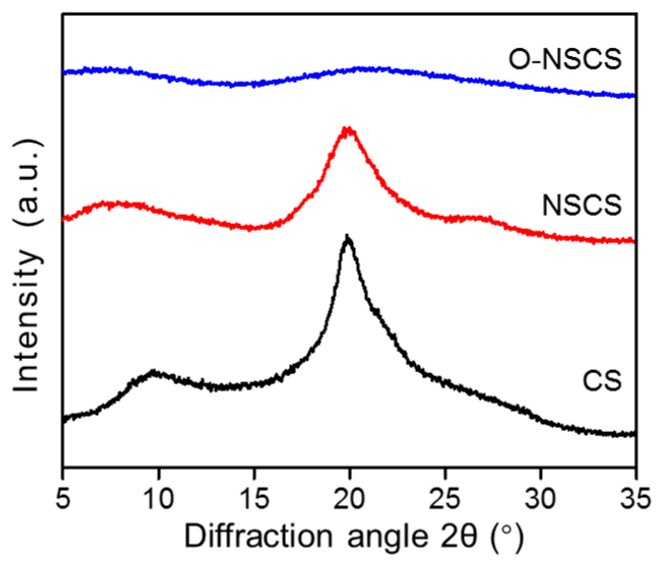
The X-ray diffraction patterns of CS, NSCS, and O-NSCS.

X-ray diffraction measurements were conducted to clarify the changes of chitosan structure before and after modification, as shown in Figure 3.

CS was found to exhibit two distinct diffraction peaks at around 10° and 20° (2θ), corresponding to (020) and (110) reflections, respectively. This can be ascribed to the strong inter- and intra-molecular hydrogen bonds between the hydroxyl and amino groups, which make chitosan form a highly ordered crystalline structure and therefore become insoluble in water. Comparatively, in the case of NSCS, the peak at 11° vanished and the peak at 20° weakened, confirming the successful succinylation of chitosan and suggesting that the introduction of succinyl groups can decrease the hydrogen bonding capacity and loosen the molecular chains of CS. As a result, NSCS can be dissolved in water and is easy to react to. Subsequently, the pattern of O-NSCS displayed broad amorphous features and no peaks indicative of crystalline phases.

#### 2.2.4. Differential Scanning Calorimeter (DSC)

To investigate the water absorption and retention properties of chitosan and its derivatives, we designed a thermo-gravimetric experiment with DSC and analytical balances. Commercial hyaluronic acid (HA) with a molecular weight of 200 kDa was employed for comparison.

**Figure 4:**
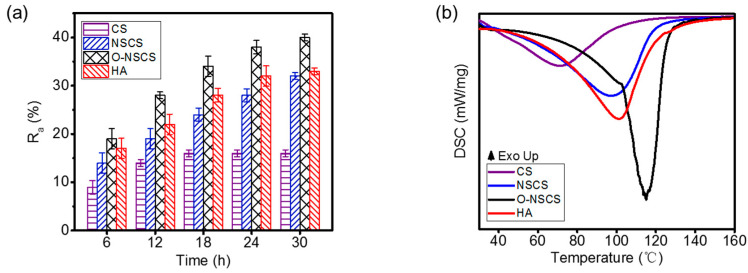
(**a**) Moisture-absorption ability of different samples at 20 °C and relative humidity 81% (repeated three times). (**b**) Differential scanning calorimeter (DSC) curves of different samples.

Figure 4a gives the percentage of weight increase in different dry samples (Ra). As shown, the water absorption property of NSCS had been improved to some extent, but, significantly, O-NSCS exhibited the best water absorption performance, even relative to that of HA. The weight increase of all samples reached equilibrium after 50 h. Figure 4b shows the DSC curves of different samples, in which the peaks represent the water loss temperature. The higher the water loss temperature, the stronger the molecular interaction between samples and water, which indicates a better water retention performance of the sample. Based on these results, it can be concluded that the moisture retention performance was O-NSCS > HA > NSCS > CS.

Taking the above observation into account, we can make an inference as follows. There were primarily three types of hydrogen bonds between water and chitosan derivatives: H-O-H···N-H, H-O-H···O-H, and H-O-H···O-C=O, the last one of which shows the strongest bond strength due to the highest electronegativity of oxygen in carboxylate groups. It is necessary to mention that the moisture absorption is closely related to the hydrogen bond capacity between water and samples, and the moisture retention is dependent on hydrogen bond strength. For CS, high crystallinity resulted in only fewer free hydroxyl and amino groups forming hydrogen bonds with water. In the case of NSCS, the destruction of the crystalline regions led to an increase in the number of free hydroxyl and amino groups. Meanwhile, the introduction of carboxyl groups made it possible to form a stronger hydrogen bond. Consequently, the water absorption and retention performance of NSCS were better than that of CS. Furthermore, the amorphous structure of O-NSCS and the increase in carboxyl groups led to an improvement of moisture absorption and retention abilities.

## 3. Materials and Methods

### 3.1. Materials

Commercial CS was obtained from Sigma-Aldrich (Beijing, China). The viscosity-average molecular weight (Mv) of 1000 kDa and the deacetylation degree (DD) of 88% were determined by the Mark–Houwink–Sakurada equation and ^1^H NMR spectroscopy [24]. Succinic anhydride (C_4_H_4_O_3_, assay >99%, SA) was furnished by Beijing InnoChem Science & Technology Co., Ltd. (Beijing, China). TEMPO (assay >98%) was purchased from Acros Organics (Shanghai, China). Sodium hypochlorite pentahydrate (NaClO·5H_2_O) was obtained from TCI Shanghai (Shanghai, China). Acetone (CH_3_COCH_3_) was of analytical grade and furnished by Changlian Chemical Reagent Industries, Ltd. (Chengdu, China). Chemicals dimethyl sulfoxide (DMSO), sodium hydroxide (NaOH), sodium bromide (NaBr), hydrochloric acid (HCl), and anhydrous ethanol (CH_3_CH_2_OH) were of analytical grade and purchased from Chengdu Kelong Chemical Co., Ltd. (Chengdu, China). Chitosan was ground by a Baijie BJ-200 stainless steel grinder to pass through a 100-mesh sieve and used for further study. Other reagents were used without further purification.

### 3.2. Methods

#### 3.2.1. Preparation of *N*-Succinyl Chitosan

*N*-succinyl chitosan was prepared according to the process in Yan et al. [25], with some modification. Pretreated chitosan powder (5.00 g) was suspended in DMSO (100 mL), and succinic anhydride (5.00 g) was added to the suspension. This was followed by stirring at 500 rpm while maintaining the temperature at 60 °C. After standing for 4 h, the solvent was removed by filtration, and the solid left was collected and dispersed in 400 mL of H_2_O, whose pH was adjusted to 10~11 with 5% (*w*/*v*) NaOH solution to form a pale-yellow solution. The crude product was precipitated by adding acetone of three times as much as the volume of the solution, and washed alternatively with 75% ethanol and 70% acetone four times. The wet acylation product was then washed with pure acetone before being dried in vacuo at 60 °C for 48 h. The obtained pale-yellow powder was NSCS.

#### 3.2.2. Oxidation of *N*-Succinyl Chitosan Mediated by TEMPO

C-6 specific oxidation of NSCS was performed based on the oxidation of chitin by the TEMPO/NaClO/NaBr system [7,9]. NaBr (0.4 mol per mol of the glycosidic unit) and a solution containing 5% NaClO (2.0 mol NaClO per mol of glycosidic unit) were added into 2.0% NSCS aqueous solution, which was stirred at 0–5 °C. Immediately after the introduction of NaClO, the pH was adjusted to 10.8 with 1.2 mol/L HCl aqueous solutions. TEMPO (0.01 mol per mol of the glycosidic unit) dissolved in 10 mL H_2_O was then added to the solution, which was the starting point of the reaction. The pH was maintained at 10.8 by the continuous addition of 0.5 mol/L NaOH aqueous solution and the volume of NaOH solution added was monitored. Ethanol (3.0 mL per gram of NSCS) was added to the solution to quench the oxidation. The solution was then neutralized with dilute HCl and dialyzed through a dialysis membrane (molecular weight cut off, 8000; Yuanye biotechnology Co., LTD., Shanghai, China) for three days until no Cl^−^ was detected, and then lyophilized to yield a white powder, which was 6-oxidized *N*-succinyl chitosan (O-NSCS). A “blank” reaction was also performed with all the reactants added, but in the absence of TEMPO.

#### 3.2.3. FT-IR Spectroscopy

Fourier Transform Infrared (FT-IR) spectroscopy of the native and modified chitosan was carried out on a Bruker Tensor 27 FT-IR spectrometer (Bruker, Karlsruhe, Germany) using KBr pellets. Spectra were collected over the range 4000–400 cm^−1^ at room temperature.

#### 3.2.4. NMR Spectroscopy

^1^H and ^13^C NMR spectra of chitosan and its derivatives were recorded on a Bruker VARIAN III 600MHz spectrometer (Bruker, Zurich, Switzerland), using tetramethyl silane (TMS) as an internal standard. Chitosan was dissolved in 0.1% DCl, while NSCS and O-NSCS were characterized using D_2_O as a solvent.

#### 3.2.5. Determination of the Degree of Oxidation (DO)

During the oxidation process, the pH dropped due to the conversion of a primary alcohol to carboxylic acid, which was titrated by NaOH. The consumption of NaOH was proportional to the amount of oxidized primary alcohol. Therefore, the amount of NaOH consumed could be used to calculate the DO [21]. The equation for a calculation is shown in Equation (2):

(2) $$\mathrm{DO}=V\times C\times \frac{M}{m}\times 100\%$$

where *V* (dm^3^) is the volume of NaOH solution added; C (mol/L) is the accurate concentration of NaOH aqueous solution determined by titration; M is the average relative molecular mass of the glycoside units in NSCS; *m* (g) is the weight of NSCS.

The desired DO value of products could be obtained by calculating the theoretical consumption of NaOH solution and quenching the reaction in time.

#### 3.2.6. X-ray Diffraction (XRD)

X-ray diffraction patterns were collected from 5° to 35° (2θ) using X’Pert Pro MPD DY129 (Malvern Panalytical B.V., Almelo, Netherlands), employing Cu Kα filtered radiation (λ = 0.154 nm) at 40 kV and 35 mA.

#### 3.2.7. Differential Scanning Calorimeter (DSC)

The DSC thermograms were recorded on a DSC 200PC (NETZSCH co., Selb, Germany) to investigate the interaction of chitosan derivatives with water. The measurements were performed under a nitrogen atmosphere from 30 to 160 °C at a heating rate of 10 K/min. Before the DSC testing, the dried samples were placed in a desiccator containing a saturated aqueous (NH_4_)_2_SO_4_ (relative humidity = 81%) to determine the water absorption ability, which was evaluated by the percentage of weight increase in dry sample (R_a_) as shown in Equation (3):

(3) $$R_{a}=\frac{W_{n}-W_{0}}{W_{0}}\times 100\%$$

where *W*_0_ and *W_n_* are the weights of the sample before and after putting it into the desiccator.

## 4. Conclusions

In summary, the *N*-succinylation disrupted the crystalline phases of chitosan and resulted in a great improvement of the water solubility, moisture absorption and retention abilities. The moisture absorption and retention properties of NSCS determined by gravimetric methods and DSC were close to that of commercial HA. Furthermore, NSCS was proven to be a good substrate to conduct C-6 specific oxidation mediated by the TEMPO/NaOCl/NaBr system. Water solubility allowed the oxidation to be carried out under homogeneous conditions, ensuring a high reaction rate, and the ^13^C NMR spectrum showed a high selectivity and conversion rate. While a detailed mechanism of water retention property remains to be further investigated, the oxidation products of NSCS can be considered as a new polysaccharide exhibiting great water absorption and retention abilities, even better than that of HA. The abundant reserves and low price of raw materials also mean this new class of polysaccharides has a promising prospect in cosmetics and medical applications. Besides, we should make efforts to find a pre-modification approach or develop a novel, effective, and environmentally friendly oxidation system that can be applied to chitosan to avoid degradation.
